# What is synergy? The Saariselkä agreement revisited

**DOI:** 10.3389/fphar.2015.00181

**Published:** 2015-09-01

**Authors:** Jing Tang, Krister Wennerberg, Tero Aittokallio

**Affiliations:** Institute for Molecular Medicine Finland (FIMM), University of HelsinkiHelsinki, Finland

**Keywords:** definition of synergy, drug combinations, Bliss and Loewe models, interaction barometer, consensus agreement

## Abstract

Many biological or chemical agents when combined interact with each other and produce a synergistic response that cannot be predicted based on the single agent responses alone. However, depending on the postulated null hypothesis of non-interaction, one may end up in different interpretations of synergy. Two popular reference models for null hypothesis include the Bliss independence model and the Loewe additivity model, each of which is formulated from different perspectives. During the last century, there has been an intensive debate on the suitability of these synergy models, both of which are theoretically justified and also in practice supported by different schools of scientists. More than 20 years ago, there was a community effort to make a consensus on the terminology one should use when claiming synergy. The agreement was formulated at a conference held in Saariselkä, Finland in 1992, stating that one should use the terms Bliss synergy or Loewe synergy to avoid ambiguity in the underlying models. We review the theoretical relationships between these models and argue that one should combine the advantages of both models to provide a more consistent definition of synergy and antagonism.

## Introduction

Evaluation of interaction effects between biologically active agents has become an important topic in many disciplines, including pharmacology (Cokol et al., 2011; Miller et al., 2013; Chevereau and Bollenbach, 2015), biochemistry (Hu et al., 2011; Zhang and Viikari, 2014; Bunterngsook et al., 2015), and environmental sciences (Darling and Côté, 2008; Piggott et al., 2015). The interaction between multiple agents is often classified as either synergistic or antagonistic, depending on how much the observed combination response differs from the expected response under the null hypothesis that the two agents are non-interacting. Multiple reference models have been formulated based on a distinctive set of empirical or biological assumptions (see e.g., Lehár et al., 2009). These assumptions, albeit difficult to validate a priori due to the lack of precise knowledge of the mechanisms of action, are often justifiable as long as they provide biologically plausible reasoning about the nature of non-interaction. However, the inherent differences in the model assumptions have inevitably led to inconsistency in the quantification of the degree of interaction, contributing to a major source of confusion and controversy on the definitions of synergy and antagonism. The Saariselkä agreement, proposed more than 20 years ago, aimed at reaching a consensus on the terminology for characterizing the degree of interaction (Greco et al., 1992). Acknowledging the theoretical background of the major competing models, the Saariselkä agreement admitted that there is no single universally best reference model. Rather than continuing the debate on the appropriateness of the model assumptions, the agreement called for a compromise between the advocates of the models, and proposed a practical guideline for reporting synergy or antagonism, where the underlying reference model should be explicitly described to avoid any ambiguity.

## The two reference models

For the rest of the review, we will use drug combination as an example of interaction data. A drug's effect *y* is often measured at a certain dose *x* as the percentage of biological response, i.e., *x* > 0, 0 < *y* < 1. Let us consider that, drug 1 at dose *x*_1_ produces a response *y*_1_ and drug 2 at dose *x*_2_ produces a response *y*_2_. Next, we combine the two drugs at the dose pair (*x*_1_, *x*_2_) and observe a combination response *y*_*c*_. To quantify the degree of drug interaction, we need to formulate a reference model to answer the following question: if there is no interaction between the drugs, what would be the expected combination response *y*_EXP_ at (*x*_1_, *x*_2_)? So far, two major reference model classes have been proposed, the Bliss independence model (Bliss, 1939) and the Loewe additivity model (Loewe, 1953). While each having its own logical basis, the underlying assumptions behind these two models are relatively distinct.

The Bliss independence model adopts a probabilistic perspective by treating a drug combination under non-interaction as a joint action of independent, yet competing perturbations by the individual drugs. Such a probabilistic independence allows the expected combination response to be computed as the product of the individual drug responses:

(1) $$\begin{array}{l} y_{\text{BLISS}}=y_{1}+\left(1-y_{1}\right)y_{2}=y_{1}+y_{2}-y_{1}y_{2}. \end{array}$$

An observed combination response greater or smaller than *y*_BLISS_ can be interpreted as a departure from the probabilistic independence, which thus implies an interaction between the two drugs. The Loewe additivity model, on the other hand, requires additional information about the dose-response relationships of the individual drugs. Namely, let *y* = *f*_1_(*x*) and *y* = *f*_2_(*x*) be the dose-response functions for drug 1 and drug 2, respectively. Then the doses at which each drug alone produces the expected response *y*_LOEWE_ can be represented as, $f_{1}^{-1}\left(y_{\text{LOEWE}}\right)$ and $f_{2}^{-1}\left(y_{\text{LOEWE}}\right)$, where *f*^−1^ is an inverse function which maps the response *y* back to the dose *x*. Formally, the Loewe additivity model states that *y*_LOEWE_ must satisfy:

(2) $$\begin{array}{l} \frac{x_{1}}{f_{1}^{-1}\left(y_{\text{LOEWE}}\right)}+\frac{x_{2}}{f_{2}^{-1}\left(y_{\text{LOEWE}}\right)}=1. \end{array}$$

The rationale behind Equation (2) is to fit non-interaction to the so-called sham experiment scenario, where a drug is combined with itself, that is, *f*_1_(*x*) = *f*_2_(*x*). According to Equation (2), one can derive *y*_LOEWE_ = *f*(*x*_1_ + *x*_2_) for the sham experiment, reflecting the intuition that combining two drugs of the same type should induce neither synergy nor antagonism.

## The saariselkä agreement

The assumptions and performance of the two reference models have been compared and discussed in many review articles (e.g., Berenbaum, 1989; Greco et al., 1995; Chou, 2006; Lee, 2010; Zhao et al., 2010). There have been attempts to distinguish the Bliss and Loewe models in terms of mechanistic implications (Shafer et al., 2008; Laskey and Siliciano, 2014; Chevereau and Bollenbach, 2015). The Bliss independence model is expected to hold true for non-interacting drugs that elicit their responses independently, e.g., by targeting separate pathways. Loewe additivity, in contrast, is more compatible with the case where the drugs have similar modes of action on the same targets or pathways. However, little is known about whether such mechanistic justifications for the Bliss and Loewe models reflect the reality. Further, with increasing understanding of drugs' modes of action, any “previously unexpected” interaction effect becomes more expected, which makes the reference models totally dependent on the temporal state of knowledge. As pointed out in the Saariselkä agreement (Greco et al., 1992), and also by many others, neither Loewe additivity nor Bliss independence is necessarily reflecting the expected modes of action of a drug combination (Fitzgerald et al., 2006; Yeh et al., 2006; Breitinger, 2012). Rather, Loewe and Bliss models should be used as data exploratory approaches, with a major purpose to identify potential synergistic drug combinations that warrant further mechanistic investigation, but not the other way around, i.e., using the mechanistic evidence to determine which reference model is more appropriate.

After reaching the common understanding on the model assumptions, the Saariselkä agreement allowed the researchers for the flexibility to choose a preferred reference model to evaluate interactions of multiple agents, with the only precondition that the names of the specific models need to be explicitly reported. Namely, depending on which model is used, a combination response greater or less than *y*_EXP_ will be termed as Loewe synergy, Loewe antagonism, Bliss synergy or Bliss antagonism, respectively. Following these recommendations, the controversy over the definitions of synergy and antagonism seemed subsided. More recently, a variety of interaction assessment methods have been further developed and applied to a wide range of biological research fields. Notably, most of these methods can be traced back to the two basic model classes. For example, variants of the Loewe additive model include combination index (Lee et al., 2007; Chou, 2010), isobologram analysis (Tallarida, 2006) and response surface models (Greco et al., 1995; Kong and Lee, 2006); variants of the Bliss independence model include various synergy contour approaches (Fitzgerald et al., 2006; Zhao et al., 2014).

## What is synergy?

Paradoxically, even with the clear distinction that has been made between the reference models, we feel that the fundamental question still remains unanswered, if not becoming even more serious: What is synergy after all? Since the expected combination responses *y*_BLISS_ and *y*_LOEWE_ most often are not identical (Berenbaum, 1989), choosing the model to use has become a practical burden for a researcher who tries to draw solid biological conclusions out from the data. Due to the lack of practical guidelines, the model selection has become a personal preference or largely a convention that has been followed in a particular research field without clear reasons (Lee et al., 2007; Zhao et al., 2014). There has been a tendency to favor a model that yields a lower expected combination response, as it results in a higher likelihood of detecting stronger synergy. To make matters even worse, there has been often a dilemma when a drug combination is classified as synergistic according to one model but antagonistic according to the other (Cokol et al., 2011). The Saariselkä agreement, unfortunately, seem to have failed to provide any recommendations for solving these practical issues. What the Saariselkä agreement achieved was a compromise for accepting individualized claims, but the ultimate aim to advance the consensus knowledge on the degree of interaction has remained largely missing.

**Figure 1:**
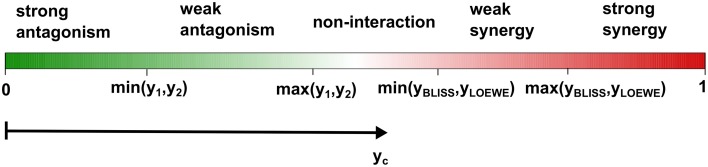
**The proposed terminology for classifying drug interactions**. Using the interaction barometer allows a direct comparison between different drug combinations in terms of their degrees of interaction as well as their combination responses. If the observed drug combination effect *y*_*c*_ is lower than the maximum single drug effect max(*y*_1_, *y*_2_) but higher than the minimum effect min(*y*_1_, *y*_2_), then the combination is called weak antagonism; if *y*_*c*_ < min(*y*_1_, *y*_2_) it is called strong antagonism. To classify synergy, we consider the Bliss and Loewe models, with the expected effects denoted as *y*_BLISS_ and *y*_LOEWE_, respectively. If max(*y*_1_, *y*_2_) < *y*_*c*_ < min(*y*_BLISS_, *y*_LOEWE_), then we call the combination as non-interaction; if *y*_HSA_ < *y*_*c*_ < min(*y*_BLISS_, *y*_LOEWE_) it is called weak synergy, and for *y*_*c*_ > max(*y*_BLISS_, *y*_LOEWE_) strong synergy.

To ease the model selection burden, we propose here the use of new terminology that incorporates both of the two reference models, together with the single drug responses, to distinguish non-interaction, synergy and antagonism. With simple algebra, one can show that max(*y*_1_, *y*_2_) ≤ *y*_BLISS_. For the Loewe additivity model with a monotonically increasing dose-response relation, one can also show that max(*y*_1_, *y*_2_) ≤ *y*_LOEWE_. We note that, max(*y*_1_, *y*_2_) is also the expected response from a popular reference model, called highest single agent (HSA) model (Berenbaum, 1989). If the combination response *y*_*c*_ is lower than max(*y*_1_, *y*_2_), then one would intuitively infer antagonism. Therefore, we may use max(*y*_1_, *y*_2_), to distinguish antagonism from non-interaction. Similarly, one can use the response of the less effective single drug, that is min(*y*_1_, *y*_2_), to further distinguish between weak and strong antagonisms. For distinguishing synergy from non-interaction the answer is less obvious, as it depends on the comparison between *y*_BLISS_ and *y*_LOEWE_. There has been considerable interest in the mathematical relationships between the Bliss independence and the Loewe additivity models to understand how much difference in the characterization of drug interaction one can expect when choosing one model over another (see e.g., Goldoni and Johansson, 2007). In particular, two authors of the Saariselkä agreement have reported results from such comparisons (Drescher and Boedeker, 1995; Dressler et al., 1999). They showed that *y*_LOEWE_ > *y*_BLISS_ is generally observed for very steep dose-response curves, while *y*_LOEWE_ < *y*_BLISS_ when the curves become more flat. Since, *y*_BLISS_ and *y*_LOEWE_ differ in a complex way depending on the parameterization of the dose-response functions, we propose two cut-offs, min(*y*_BLISS_, *y*_LOEWE_) and max(*y*_BLISS_, *y*_LOEWE_), for characterizing synergistic combinations. We reason that the consistency between the Bliss independence and the Loewe additivity models should be indicative of the degree of synergy: If both the Bliss model and the Loewe model classify a drug combination as synergistic, that is, *y*_*c*_ > max(*y*_BLISS_, *y*_LOEWE_), then we call it a strong synergy; If the combination is classified as synergistic according to one model only, that is, min(*y*_BLISS_, *y*_LOEWE_) < *y*_*c*_ < max(*y*_BLISS_, *y*_LOEWE_), then it is called weak synergy. Finally, non-interacting drugs have max(*y*_1_, *y*_2_) < *y*_*c*_ < min(*y*_BLISS_, *y*_LOEWE_), reflecting our view that non-interaction should also be defined similarly as a range, rather than a single point as in the individual reference models. Given such a classification, one may continue to develop statistical testing methods for evaluation of its significance for replicate data. To facilitate better understanding of these definitions, we designed an interaction barometer that enables a systematic comparison of these proposed interaction terms along an axis of drug combination response *y*_*c*_ (Figure 1).

The benefits of adopting the proposed terminology for the degree of interaction are two-fold. First, the definitions of synergy and antagonism are based on a simultaneous evaluation of the two reference models, as well as the individual drug responses. Such a data-driven approach avoids any pre-defined preference either for the Bliss independence or Loewe additivity when characterizing drug interactions, and thus it minimizes the biases toward either of the models. This is consistent with the idea that any synergy model should be treated as an exploratory ranking statistic for prioritization of the most potent combinations for further evaluation, rather than a “true model” for explaining synergy or antagonism mechanisms. Further, this terminology enables a more intuitive definition of non-interaction, under which the combination response may be higher than the single drug response, but not as high as the expected responses from the Bliss independence and the Loewe additivity models. Note that a drug combination falling into such an interval would be classified as antagonistic according to both of the two models, but since it produces a higher response than the single drugs, one would rather characterize it as an additive effect or non-interaction. For the sake of clarity, we would call it non-interaction, and in fact discourage the terms additive or additivity since these may be confused with the additivity implicated by the Loewe additivity model. The interval of non-interaction shown in Figure 1 is positioned at the center of the barometer as a gray zone for those drug combinations with no clear evidence in support of either synergy or antagonism.

Secondly, the different interaction terms are positioned along the common response axis (e.g., measured as the percentage inhibitions of cell growth), which makes it easier to relate the degree of drug interaction with its outcome in the drug response. With the proposed interaction barometer (Figure 1), one can immediately tell the differences between the drug combination response *y*_*c*_ and the responses of individual drugs (*y*_1_, *y*_2_), as well as the expected combination responses (*y*_BLISS_, *y*_LOEWE_) based on the two reference models. The clear correspondence between the degree of synergy and the combination response is in many ways superior to the use of an interaction index, such as combination index or other similar approaches (see e.g., Lee et al., 2007; Lee, 2010), which tend to be less obvious to interpret in terms of response boosting. For example, a combination index of 0.1 has been considered as a very strong synergy by Chou (2006), but how much extra response the synergy can produce for the drug combination is difficult to tell. In contrast, with the interaction barometer one can easily visualize the levels of boosted response of the combination compared to the single drugs or reference models. From the model development perspective, the graphical representation of the competing reference models in the interaction barometer may facilitate a more systematic comparison among different approaches. For example, when new reference models are introduced, one can always position its expected combination response onto the barometer to enable better understanding of its relationships with the existing models.

## Synergy vs. Efficacy

So far, we have merely discussed about the assessment of drug interactions using the difference between the observed combination response and its theoretical expectation, i.e., *y*_*c*_ − *y*_EXP_, to classify a drug combination as synergistic or antagonistic. However, a drug combination can be also classified as either effective or ineffective based solely on its actual combination response *y*_*c*_. It is important not to confuse these two concepts, synergy and efficacy, as the nomenclatures are related but should not be treated the same. Synergy is a measure of the degree of drug interaction, while efficacy is a measure of phenotypic response of a drug combination. It is possible that a drug combination is highly synergistic, while its actual response may be insufficient to reach therapeutic efficacy. On the other hand, a drug combination that exhibits strong response does not necessarily imply a synergistic interaction. For instance, only one of its component drugs may produce the response, while the other one is simply lowering the adverse effect of the first drug without affecting its on-target activity. In preclinical testing, a drug combination with strong synergy and efficacy should be prioritized for further mechanistic investigation, with an additional requirement of tolerable toxicity profile (Fitzgerald et al., 2006). Accordingly, the dosages of a drug combination are also important factor for clinical feasibility and for maintaining acceptable side effects. For instance, the concept of therapeutic synergy compares the therapeutic windows of the single agents to that of their combinations, instead of using compound efficacies alone (Kashif et al., 2014). However, the main focus of this review was the definition of synergistic interaction, and we refer those readers interested in the therapeutic significance of synergy in drug discovery to previous reviews (Fitzgerald et al., 2006; Sucher, 2014).

## Conclusion

The definition of synergistic interaction is still under debate. After a careful investigation of the Bliss independence model and the Loewe additivity model, we argue that, without jeopardizing the validity of both models, a more consistent terminology for classifying synergy and antagonism can be made. By comparing the observed combination response with the expected combination responses from the two models, as well as the single drug responses, one can classify the drug combination into five categories including strong antagonism, weak antagonism, non-interaction, weak synergy, and strong synergy. We propose the use of the interaction barometer to visualize the degree of interaction on the common axis of drug response, which has been shown to facilitate the interpretation and comparison between different combinations. We view our efforts as a continuation to what the Saariselkä agreement started more than 20 years ago but has not yet concluded: a consensus on concepts and terminology for interaction assessment. We acknowledge that our proposal is not yet solving the practical issues for analyzing real data which typically contain combination responses tested at different dose ranges. How to maximize the benefits of the interaction barometer to summarize the interaction patterns of a drug combination would be a source of future research initiatives. We hope that such a classification scheme will raise more discussions about the standardization of the interaction assessment, toward finally reaching a consensus not only on the definition itself, but also on the other important issues, such as the experimental design of combination experiments, their quality control and the statistical evaluation of synergy and antagonism.

## Funding

This work was supported by the Academy of Finland (grants 272437, 269862, 279163, and 292611 for TA, 277293 for KW); Cancer Society of Finland (JT, TA, and KW).

### Conflict of interest statement

The authors declare that the research was conducted in the absence of any commercial or financial relationships that could be construed as a potential conflict of interest.
